# Clinical Performance of the LiquidArray^®^ Gastrointestinal VER 1.0 Assay in Patients with Suspected Gastroenteritis

**DOI:** 10.3390/diagnostics14212377

**Published:** 2024-10-25

**Authors:** Sophie Jones, Kathleen Pheasant, Colette Dalton, Julie Green, Catherine Moore

**Affiliations:** 1 Bacteriology Department, Public Health Wales Microbiology Cardiff, University Hospital of Wales, Health Park, Cardiff CF14 4XW, UK; sophie.jones2@wales.nhs.uk; 2 Wales Specialist Virology Centre, Public Health Wales Microbiology Cardiff, University Hospital of Wales, Health Park, Cardiff CF14 4XW, UK; kathleen.pheasant@wales.nhs.uk; 3 Bruker Microbiology and Infection Diagnostics, Bruker UK Limited, Nova Business Park, Glasgow G33 1AP, UK; colette.dalton@bruker.com

**Keywords:** gastroenteritis, diarrhoea, multiplex gastrointestinal panel, syndromic panel, molecular diagnostics, enteric pathogens, gastrointestinal infection, stool, diagnostic accuracy

## Abstract

Background/Objectives: Rapid and accurate pathogen identification is essential for the proper management of patients with infectious gastroenteritis, as well as for a better control of disease outbreaks. This observational, non-interventional, single-site study evaluated the diagnostic accuracy of LiquidArray^®^ Gastrointestinal VER 1.0, a multiplex PCR syndromic panel capable of detecting up to 26 clinically relevant enteropathogens. Methods: Sensitivity, specificity, positive predictive value (PPV), negative predictive value (NPV) and likelihood ratio (LR) were evaluated using stool samples from 1512 patients with suspected gastroenteritis and were compared to seven competitor assays. Results: LiquidArray^®^ Gastrointestinal VER 1.0 showed a very low invalid rate (0.5% at initial testing, down to 0% after repeat) and high sensitivity (>90% for most detected targets) and specificity (>99% for all detected targets). Accordingly, the PPV and NPV were high (>90% for most targets and >99% for all targets, respectively). The analytical performance of LiquidArray^®^ Gastrointestinal VER 1.0 was also excellent as to co-amplification capability, cross-reactivity and assay precision. Conclusions: This study demonstrates the excellent clinical performance of LiquidArray^®^ Gastrointestinal VER 1.0 and its suitability for implementation in clinical routine for the rapid and accurate diagnosis of infectious gastroenteritis.

## 1. Introduction

Infectious gastroenteritis is a major cause of morbidity and mortality worldwide, especially in young children, the elderly and immunocompromised patients [1,2]. Diagnosis and management guidelines assist healthcare professionals in the identification of enteric pathogens and the implementation of specific therapy and control measures [1,2,3,4,5,6]. A wide range of enteric pathogens, including bacteria, viruses and parasites, cause infectious gastroenteritis [1,7,8,9,10,11,12,13,14,15,16,17,18,19,20]. Early identification of the causative pathogen(s) is key to adequate patient management and the choice of appropriate targeted therapy. It is also essential to prevent infectious disease outbreaks [1,3,4]. Rapid and accurate pathogen identification remains challenging in clinical laboratory routine. Conventional diagnostic techniques, such as stool culture, biochemical and immunological testing, and microscopic examination of diarrhoeal stool specimens, are time-consuming, labour-intensive and often inaccurate [19,21]. In past years, molecular diagnostic assays based on polymerase chain reaction (PCR) enabled the rapid and accurate detection of single pathogens [19]. More recently, multiplex PCR opened a new era of comprehensive syndromic detection of clinically relevant enteropathogens. These multiplex PCR gastrointestinal (GI) panels allow the simultaneous detection of microorganisms not readily diagnosable by clinicians in the pre-multiplex era. Moreover, they demonstrate high sensitivity and specificity and reduced turnaround time to results compared to conventional methods [19,21,22,23,24,25,26,27,28,29,30,31,32,33,34,35,36,37,38,39,40,41,42]. Despite their demonstrated superior performance, multiplex PCR assays present a few limitations compared to conventional culture-based methods. Notably, they are limited to the specific targets included on the panel and they detect nucleic acid from both living and dead organisms, rendering the diagnosis of an active infection more challenging [43]. Although these syndromic panels can increase laboratory costs compared to conventional methods, several studies have shown that their implementation results in an overall reduced healthcare cost, especially when combined with an antimicrobial stewardship program [43,44].

Many multiplex PCR GI panels, several of which are already FDA-approved, have been commercialised. They differ in the number of detected pathogens, the underlying technology platform, their level of automation, their throughput capability, the hands-on processing time and the turnaround time to result [19,21,24,28,32,45,46,47].

In the landscape of multiplex PCR GI panels, the CE-IVD LiquidArray^®^ Gastrointestinal VER 1.0 assay is a qualitative, semi-automated in vitro diagnostic assay for the detection in stool specimens of 25 nucleic acid targets covering up to 26 bacterial, viral and parasitic enteropathogens and associated toxins, namely: Adenovirus 40/41, Astrovirus, Norovirus GI and GII, Rotavirus, Sapovirus, *Ascaris* spp., *Cryptosporidium* spp., *Cyclospora cayetanensis*, *Entamoeba histolytica*, *Giardia lamblia* (also known as *Giardia duodenalis*), *Campylobacter* spp., *Clostridioides difficile* toxins A and B, *Escherichia* (*E.*) *coli* O157 gene, eae gene (found in enteropathogenic *E. coli* (EPEC) and enterohemorrhagic *E. coli* (EHEC)), enteroinvasive *E. coli* (EIEC)/*Shigella* spp., enteroaggregative *E. coli* (EAEC), enterotoxigenic *E. coli* (ETEC), *Plesiomonas shigelloides*, *Salmonella* spp., Shiga toxin genes *stx1*/*stx2* (found in Shiga toxin-producing *E. coli* (STEC) and enterohemorrhagic *E. coli* (EHEC)), *Vibrio* spp., *Vibrio cholerae* and *Yersinia enterocolitica* (Table S1). As many as 48 samples can be processed and analysed at once in about five hours and less than one-minute hands-on time per sample (45 min for 48 samples). The assay integrates automated nucleic acid extraction, PCR amplification and software-guided analysis, providing results at a glance with no need for user interpretation (Figure S1). The assay is based on the innovative LiquidArray^®^ technology, which combines real-time PCR and melting curve analysis. The LiquidArray^®^ technology was previously validated for the diagnosis of drug-resistant tuberculosis [48].

The present study aimed to validate the clinical performance of the LiquidArray^®^ Gastrointestinal VER 1.0 assay using stool samples from 1512 patients with suspected gastroenteritis collected at one centre (Public Health Wales, Cardiff, UK). Diagnostic accuracy was determined against seven CE-IVD comparator and reference methods. The LiquidArray^®^ Gastrointestinal VER 1.0 assay showed high diagnostic accuracy for most of the 25 evaluated nucleic acid targets and very low invalid rates.

## 2. Materials and Methods

### 2.1. Study Samples

Stool samples were collected from community and hospitalised patients presenting with diarrhoea and submitted to the Cardiff Public Health Wales (PHW) Microbiology Department, University Hospital of Wales (Cardiff, UK). Both prospectively collected fresh samples and archived samples collected at the same study site and stored frozen at −80 °C were included in the study. Fresh and frozen stool samples were considered to be equivalent as to pathogen detection, based on the existing literature [49,50]. In addition, contrived samples were used in case of analytes with low positive rates (very rare pathogens). Contrived samples were negative stool specimens spiked with certified pathogen material or with nucleic acids from pathogens or target sequences. Following collection, stool samples were characterized according to the Bristol Stool Chart [51], added to Stool Buffer VER 1.0, then transported and stored according to study plan (see Section 2.2).

The study was conducted in accordance with the Declaration of Helsinki, the principles of Good Clinical Practice (GCP), and the In Vitro Diagnostic Medical Devices Regulation (IVD Regulation (EU) 2017/746). Patient consent and ethics approval were waived for this study due to the use of anonymized leftover clinical materials collected as part of routine standard of care, according to local regulations.

### 2.2. Study Design

This observational, non-interventional, single-site study aimed at determining the diagnostic accuracy of the LiquidArray^®^ Gastrointestinal VER 1.0 panel using stool samples from patients with suspected gastroenteritis or indicated for excluding infectious gastroenteritis. Additional inclusion criteria were: (1) availability of sample information and of anonymized patient information, (2) sample transport to study site at room temperature within 2 days of collection, (3) sample storage until testing at 2–8 °C for no longer than 5 days after collection and longer storage (>5 days) at −80 °C, (4) time interval between LiquidArray^®^ Gastrointestinal and comparator method testing <48 h, (5) no more than two samples from the same patient and (6) sufficient stool material for three nucleic acids extractions. Exclusion criteria were: (1) missing LiquidArray^®^ Gastrointestinal result, (2) missing comparator result, (3) missing reference method result (when applicable), (4) samples not processed and analysed according to study plan and (5) unreliable result due to operational error.

LiquidArray^®^ Gastrointestinal results were compared to results of comparator methods (Table 1). Seven CE-IVD competitor assays were used to cover the panel of 25 targets included in the LiquidArray^®^ Gastrointestinal assay (Allplex GI-Bacteria(I), Allplex GI-Bacteria(II), Allplex GI-Viral, Allplex GI-Parasite and Allplex GI-Helminth(I) from Seegene, Seoul, South Korea; RealStar^®^ Clostridium difficile PCR Kit 2.0 from Altona Diagnostics, Hamburg, Germany; and BioFire^®^ FilmArray^®^ GI Panel from BioFire Diagnostics, Salt Lake City, UT, USA). Each comparator method is intended to detect one to eight of these 25 targets (Table 1).

In case of discordance between the LiquidArray^®^ Gastrointestinal and comparator results, a reference method was additionally applied (BioFire^®^ FilmArray^®^ Gastrointestinal Panel), except for the detection of *P. shigelloides* and *V. cholerae*, whose comparator method was already the BioFire^®^ FilmArray^®^ Gastrointestinal Panel, and for the detection of *Ascaris* spp. (Table 1). This approach aimed to reduce a potential bias associated with the comparator methods and hence improve the robustness of the performance analysis. Accordingly, this performance evaluation study compared the results of LiquidArray^®^ Gastrointestinal VER 1.0 to those of the comparator method. In case of discordant LiquidArray^®^ Gastrointestinal vs. comparator results, the sample was tested by the reference method, with results evaluated using the two-by-three-rule, meaning that a sample was rated as a true positive if two of three assays yielded a positive result.

Performance parameters included diagnostic sensitivity, diagnostic specificity, positive predictive value (PPV), negative predictive value (NPV) and likelihood ratio (LR). The rate of indeterminate and invalid results before and after repeat testing was also calculated. To evaluate the diagnostic performance of detection of the very rare pathogens *P. shigelloides*, *V. cholerae* and *Ascaris* spp., a subset of the prospective sample collection was tested with the methods of comparison to determine statistically significant values for diagnostic specificity (see Section 2.5). To examine the diagnostic sensitivity of detecting these rare pathogens, contrived samples were used instead.

### 2.3. LiquidArray^®^ Gastrointestinal Method

LiquidArray^®^ Gastrointestinal VER 1.0 is a CE-IVD assay approved under the EU In Vitro Diagnostic Medical Device Regulation (IVDR). It is indicated as an aid for diagnosis and intended for use by laboratory professionals and diagnostic laboratories. LiquidArray^®^ Gastrointestinal VER 1.0 is based on a combination of real-time PCR technology and melting curve analysis. It is a highly multiplexed PCR test designed to detect 25 targets (covering 26 relevant enteropathogens and associated toxins) in a 2-PCR format (PCR1 and PCR2; Table S1). It allows the analysis and interpretation of 48 stool samples in about 5 h.

LiquidArray^®^ Gastrointestinal VER 1.0 assays were performed according to instructions for use (IFU, version VER 1.0; [52]). Stool samples were stabilized in Stool Buffer and nucleic acids were extracted using the GXT96 X3 Extraction Kit on the GenoXtract^®^ fleXT automated instrument (Bruker UK/Hain Lifescience GmbH, Nehren, Germany). Following PCR setup on the GenoXtract^®^ fleXT device, real-time PCR and/or melting curves were generated on the FluoroCycler^®^ XT thermocycler (Bruker UK/Hain Lifescience GmbH). Acquired fluorescence signatures were interpreted with the FluoroSoftware^®^ XT-IVD software (version 1.0.1.5.4.73) and displayed in the FluoroCycler^®^ Report (Figures S1 and S2).

Results were interpreted as either (i) positive (nucleic acid of the respective pathogen or gene was detected), (ii) negative (nucleic acid of the respective pathogen or gene was not detected), (iii) indeterminate (the presence of nucleic acid of the respective pathogen or gene could not be determined due to either too low nucleic acids concentration or too low quality of the fluorescence signature) or (iv) invalid (the fluorescence signature of the patient sample and/or of the negative/positive controls did not meet the acceptance criteria). An invalid fluorescence signature could be due to amplification inhibitors and/or a manipulation error. According to IFU, it is optional to repeat an indeterminate result, while it is recommended to repeat an invalid result. For this study, indeterminate and invalid test results were repeated to calculate the initial and after-repeat indeterminate and invalid rates. However, initial indeterminate results and after-repeat invalid results were considered for the performance evaluation of the assay, adhering to IFU’s recommendations.

### 2.4. Comparator and Reference Methods

Competitor assays (Table 1) were performed according to the respective manufacturer’s recommendations.

For pathogen detection using the Allplex GI-Bacteria(I), Allplex GI-Bacteria(II), Allplex GI-Viral, Allplex GI-Parasite and Allplex GI-Helminth(I) assays, nucleic acids were extracted on the Microlab STARlet Automated Liquid Handler (Hamilton, Bonaduz, Switzerland) using the STARMag 96 × 4 Universal Cartridge Kit (Seegene, Seoul, Korea). PCR amplification and pathogen detection were performed using a CFX96 Real-Time PCR Detection System (Bio-Rad, Hercules, CA, USA).

For the detection of *C. difficile* toxins A and B using the RealStar^®^ Clostridium difficile PCR Kit 2.0, nucleic acids were extracted on the Microlab STARlet Automated Liquid Handler (Hamilton, Bonaduz, Switzerland) using the STARMag 96 X 4 Universal Cartridge Kit (Seegene, Seoul, South Korea). PCR amplification was performed using an Applied Biosystems 7500 Real-Time PCR System (ThermoFisher Scientific, Waltham, MA, USA).

For the detection of *P. shigelloides* and *V. cholerae*, the automated BioFire^®^ FilmArray^®^ GI Panel, which integrates sample preparation, nucleic acids extraction, multiplex PCR amplification and analysis, was used according to the manufacturer’s recommendations.

### 2.5. Analytical Performance Testing

#### 2.5.1. Precision Experiments

The precision of LiquidArray^®^ Gastrointestinal VER 1.0 was determined by repeatability and reproducibility testing of 15 key microorganisms: EPEC (eaeA+), EIEC/*Shigella* spp., *Salmonella* spp. (*S. enterica* subsp. *enterica*), *Y. enterocolitica* (*Y. enterocolitica* subsp. *enterocolitica*), Norovirus GI (recombinant), Norovirus GII (recombinant), Rotavirus (strain WA), *Cryptosporidium* spp. (*C. parvum*), *Giardia lamblia* (*G. lamblia*), *Plesiomonas shigelloides* (*P. shigelloides*), *Vibrio* spp. (*V. parahaemolyticus*), EAEC, ETEC (STh+, LT+), Adenovirus 41 and *Ascaris* spp. (*A. suum*). These microorganisms were spiked into negative stool material and processed as per IFU.

The limit of detection (LOD) of each parameter was determined throughout these experiments by collecting measurements using two operators, two FluoroCycler^®^ XT instruments and 20 replicates of each analyte at eight concentrations prepared across four dilution series (each ran on a separate plate). The tested concentrations were selected on the basis of pre-analytical testing, where an estimated LOD was achieved and the eight concentrations were analysed, including concentrations above and below this value. A total of 4928 (PCR1) and 3626 (PCR2) measurements were included in precision calculations.

#### 2.5.2. Co-Amplification Testing

Co-detection of pathogens by LiquidArray^®^ Gastrointestinal VER 1.0 was evaluated using External Quality Assessment (EQA) panel samples (Gastrointestinal Diseases Programme) from the Quality Control for Molecular Diagnostics (QCMD) organisation (https://www.qcmd.org/). Swabs from either cultured or clinical material were combined in Stool Buffer prior to nucleic acids extraction. Samples were tested in triplicates with LiquidArray^®^ Gastrointestinal VER 1.0 (PCR1 or PCR2).

#### 2.5.3. Cross-Reactivity Testing

A total of 59 commensal and non-target pathogens were tested with LiquidArray^®^ Gastrointestinal VER 1.0. Tested material was either genomic DNA spiked into negative stool samples or microorganisms extracted alongside a faecal swab in Stool Buffer using the GXT96 X3 Extraction Kit on the GenoXtract^®^ fleXT instrument. Samples were tested in duplicate at the highest available concentration. In case one of the two replicate LiquidArray^®^ Gastrointestinal target turned positive, testing was repeated in five replicates. In case of confirmed false-positive detection, sequencing was performed to rule out contamination.

### 2.6. Statistical Analyses

As for sample size estimation, a minimum of 1500 stool specimens were planned for an optimal detection of frequent and rare pathogens (i.e., up to 75 positive samples detected with the comparator method). For extremely rare pathogens (*P. shigelloides*, *V. cholerae* and *Ascaris* spp.) that are included in the LiquidArray^®^ Gastrointestinal panel, few or none of the prospectively collected and archived samples were expected to be positive for these pathogens. The minimum total sample number for extremely rare pathogens was therefore not based on the statistical significance for clinical sensitivity and positive samples but on the statistical significance for clinical specificity and the number of negative samples. To obtain highly significant data for clinical specificity, a minimum of 100 negative samples were tested in the clinical validation study. Accordingly, only a subset of the prospective sample collection (283 for *P. shigelloides* and *V. cholerae* and 110 for *Ascaris* spp.) was tested with the methods of comparison to determine the statistically significant values for diagnostic specificity. To examine diagnostic sensitivity, contrived samples (prepared from negative clinical specimens spiked with certified pathogen material or with nucleic acids) were tested under the same workflow. Thirty contrived samples were tested, including 5 positive and 25 negative for the respective pathogens.

Performance characteristics were calculated by applying the following formulas: sensitivity = TP/(TP + FN), where TP are true positives and FN false negatives; specificity = TN/(TN + FP), where TN are true negatives and FP false positives; positive predictive value (PPV) = TP/(TP + FP); negative predictive value (NPV) = TN/(TN + FN); and likelihood ratio (LR+) = sensitivity/(1-specificity). For all ratios, a 95% confidence interval (CI) was calculated using the Wilson Score method [53,54].

The precision of LiquidArray^®^ Gastrointestinal VER 1.0 was evaluated with an in-house statistics software written in Python using the statsmodels package as follows. To calculate reproducibility, an ANOVA analysis of variance was performed in combination with a linear mixed effects model. The test result (positive, negative, indeterminate or invalid) was used as the response variable in the model. The interaction between the target and the respective factors (operator, cycler, plate/run) was taken into account in the statistical model. Reproducibility results (between-operator, between-cycler and between-run precision) were expressed as variance (%), together with the respective *p*-value. *p*-values ≤ 0.05 were considered statistically significant. To calculate repeatability, the results were evaluated for all replicates of a sample at each of the eight concentrations of a dilution series. Repeatability was expressed as the probability of obtaining the same result and given together with a 95% confidence interval calculated using the binomial test method.

## 3. Results

### 3.1. Patients’ and Samples’ Characteristics

**Figure 1:**
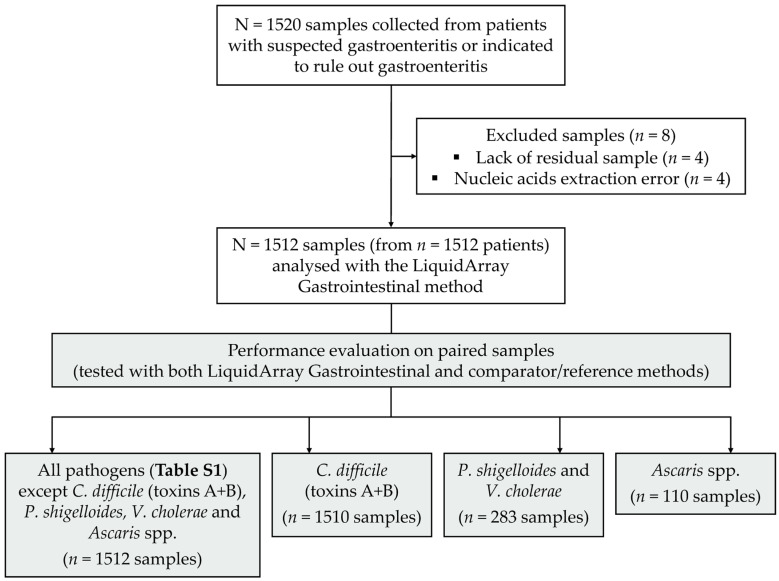
Study flow diagram. Out of the 25 tested pathogens (Table S1), 20 were evaluated by both LiquidArray Gastrointestinal and comparator/reference methods for all analysed samples (*n* = 1512). For *C. difficile* toxins A and B testing, 2 out of 1512 comparator data were missing (*n* = 1510 analysed paired samples). For the rare pathogens *P. shigelloides*, *V. cholerae* and *Ascaris* spp., only subsets of samples were tested with comparator methods, as described in the Materials and Methods section (*n* = 283 for *P. shigelloides* and *V. cholerae*, *n* = 110 for *Ascaris* spp.).

A total of 1520 patients presenting with diarrhoea and suspected of gastroenteritis or indicated for excluding infectious gastroenteritis were enrolled in the study. Of the 1520 collected stool samples, eight were excluded due to a lack of residual sample or a nucleic acids extraction error (Figure 1). All 1512 samples were tested with the LiquidArray^®^ Gastrointestinal VER 1.0 assay (thereafter referred to as “LiquidArray Gastrointestinal”).

The median (interquartile range) age of the included patients was 51 (25–71), 51.0% patients were female and most samples (91.4%) were classified as type 5–7 according to the Sample Bristol Stool Chart (Table 2).

Of the 25 targets included in the LiquidArray Gastrointestinal panel, 20 were also tested with the respective comparator methods (Table 1) on all 1512 included samples, namely: Adenovirus 40/41, Astrovirus, Norovirus GI and GII, Rotavirus, Sapovirus, *Cryptosporidium* spp., *C. cayetanensis*, *E. histolytica*, *G. lamblia*, *Campylobacter* spp., *E. coli* O157 gene, eae gene, EIEC/*Shigella* spp., EAEC, ETEC, *Salmonella* spp., Shiga toxin genes *stx1*/*stx2*, *Vibrio* spp. and *Y. enterocolitica* (Figure 1). For *C. difficile* toxins A and B, 2/1512 comparator data were missing, resulting in 1510 analysed paired samples (Figure 1). For the extremely rare pathogens *P. shigelloides*, *V. cholerae* and *Ascaris* spp., only subsets of the 1512 samples were tested with comparator methods, as described in the Materials and Methods (see Section 2.2 and Section 2.5), resulting in 283 analysed paired samples for *P. shigelloides* and *V. cholerae* and 110 analysed paired samples for *Ascaris* spp. (Figure 1).

### 3.2. LiquidArray Gastrointestinal Results

Of the 1512 analysed samples, 529 (35.0%) were positive for at least one pathogenic target using the LiquidArray Gastrointestinal assay (Table 3). Of the 529 positive samples, 386 (73.0%) corresponded to mono-detections and 143 (27.0%) to the co-detection of two to four targets (Table 3).

The invalid rate of the LiquidArray Gastrointestinal results was very low, ranging from 0.1% (for PCR 1) to 0.3% (for PCR 2). After repeat, none of the LiquidArray Gastrointestinal results were invalid (Table 4). In comparison, the invalid rate of comparator methods ranged from 0.0% to 4.5% at initial testing and from 0.0% to 0.9% after repeat (Table 4).

Hence, the overall invalid rate of the LiquidArray Gastrointestinal results was 0.5% (7/1512 tests) at initial testing and 0.0% (0/1512 tests) after repeat, while that of the combined comparator methods was 2.2% (172/7951 tests) at initial testing and 0.6% (45/7951 tests) after repeat (Table 4).

The number of valid results after repeat was considered for further analysis and only samples with valid results in both the LiquidArray Gastrointestinal and comparator methods were included in the clinical performance evaluation. The number of paired samples included in the analysis is shown in Table S2.

Among the analysed paired samples, the indeterminate rate of the LiquidArray Gastrointestinal results was very low, ranging from 0.0% (for 17/25 pathogens) to 0.1% (6/25 pathogens), 0.3% (1/25 pathogens) and 1.1% (1/25 pathogens) at initial testing, down to 0.0% (for 23/25 pathogens) and 0.1% (2/25 pathogens) after repeat (Table S3). Hence, the overall indeterminate rate of the LiquidArray Gastrointestinal results was 0.09% (30/33743 measurements) at initial testing and 0.01% (2/33743 measurements) after repeat (Table S3).

### 3.3. Clinical Performance of the LiquidArray Gastrointestinal Assay

The sensitivity, specificity, PPV, NPV and positive LR of the LiquidArray Gastrointestinal assay relative to comparator methods were determined on results valid with both approaches (Table 4 and Table S2). Positive and negative results were considered for performance calculations. LiquidArray Gastrointestinal results that were indeterminate at initial testing were excluded from performance evaluation. The performance results are shown in Table 5.

Of the 25 evaluated targets, 19 showed sensitivity >80%, ranging from 90% to 100% for 16 targets (Table 5). The very rare pathogens *E. histolytica*, *P. shigelloides*, *V. cholerae*, *Vibrio* spp. and *C. cayetanensis* were either not detected (*E. histolytica*, *P. shigelloides*, *V. cholerae*) or detected only once (*Vibrio* spp., *C. cayetanensis*) in our study (Table 5), preventing an accurate estimation of sensitivity. For these pathogens, the performance of LiquidArray Gastrointestinal was evaluated using contrived samples, revealing 100% positive percent agreement (5/5) for all five pathogens (Table S4).

Three pathogens displayed lower sensitivity: rotavirus (25/33 (75.8%)), EAEC (14/25 (56.0%)) and *Y. enterocolitica* (3/8 (37.5%)) (Table 5). These unexpected sensitivity results were further investigated. The eight discrepant rotavirus samples presented viral loads outside the dynamic range of the assay. These eight samples were positive for rotavirus upon repetition of LiquidArray Gastrointestinal on diluted samples. The eleven discrepant EAEC samples were attributed to sub-optimal primer and probe efficiency. The investigation of the five discrepant *Y. enterocolitica* samples revealed the presence of the *ystB* gene in four of these samples. The *ystB* gene is typically found in non-pathogenic biotype 1A strains [55,56,57], whereas LiquidArray Gastrointestinal was designed to detect pathogenic *Yersinia* via the *ail* gene, which is unique to virulent *Y. enterocolitica* strains [55,56,57]; comparator assays detect both pathogenic (biotypes 1B and 2–5) and non-pathogenic (biotype 1A) strains.

The specificity of the LiquidArray Gastrointestinal assay was excellent for all 25 evaluated targets, ranging from 99.3% to 100% (Table 5).

In line with the high assay sensitivity and specificity, the respective NPV and PPV parameters were also excellent. The NPV ranged from 99.1% to 100% for the 25 evaluated targets, and the PPV was >80% for the 22 pathogens detected by the comparator/reference assays, ranging from 90% to 100% for 18 of these 22 pathogens (Table 5).

All calculable LR values (12/25 pathogens), defined as the probability of target detection, were very high, ranged from 164.4 (for *Campylobacter* spp.) to 1499.0 (for *E. coli* O157 gene) (Table 5).

### 3.4. Analytical Performance of the LiquidArray Gastrointestinal Assay

Among the analytical performance parameters of the LiquidArray Gastrointestinal assay, co-amplification of several pathogens from one sample, cross-reactivity with other microorganisms (analytical specificity) and assay precision are critical for clinical application in patients with suspected infectious gastroenteritis.

The co-amplification of selected targets was successfully achieved for all tested samples (Table S5). No cross-reactivity was observed with any of the 59 commensal and non-target pathogens tested at high concentrations (Table S6). The reproducibility of the LiquidArray Gastrointestinal assay was high, with between-operator, between-instrument and between-run variance ranging from 0.00% to 0.66% across PCR1 and PCR2 (Table S7). ANOVA *p*-values were not statistically significant (*p* > 0.05), except for the between-run variability in PCR2 (*p* = 0.007), despite a low percentage of variance (0.66%). The within-run precision (repeatability) of the LiquidArray Gastrointestinal assay was also high, with probabilities of getting the same result of 96.9% and 96.2% for PCR1 and PCR2, respectively (Table S7). Further analytical performance analyses are reported in the LiquidArray Gastrointestinal IFU [52].

## 4. Discussion

This observational, non-interventional, single-site study evaluated the clinical performance of the LiquidArray Gastrointestinal assay against comparator methods in 1512 patients with diarrhoea suspected of having infectious gastroenteritis.

The positive rate (35.0% of all detections) and the co-detection rate (27.0% of positive detections) of the LiquidArray Gastrointestinal assay were in range with those reported in the literature for other multiplex PCR assays [27,34,35,36,38,42,44,58].

The indeterminate rate of the LiquidArray Gastrointestinal assay was very low (<0.1%), and the invalid rate was lower than that of comparator methods (0.5% vs. 2.2% at initial testing and 0% vs. 0.6% after repeat, respectively). The very low invalid rate of the LiquidArray Gastrointestinal assay is expected to have a positive impact on the overall time to result in clinical routine by limiting the number of sample repetitions, as well as on patient management, by yielding 100% valid test results.

The sensitivity and PPV of pathogen detection using the LiquidArray Gastrointestinal assay were >90% for most targets, while the specificity and NPV were >99% for all 25 targets. The diagnostic accuracy of the LiquidArray Gastrointestinal assay was hence in line with that reported for other marketed multiplex PCR gastrointestinal panels [19,23,24,28,29,32,43,59].

Of the 25 evaluated pathogens of the LiquidArray Gastrointestinal panel, rotavirus and EAEC showed a lower detection sensitivity vs. comparator methods (75.8% and 56.0%, respectively), apparently attributed to technical considerations that are currently being addressed by the manufacturer and are expected to be revised in a future version of the assay. The lower detection sensitivity of *Y. enterocolitica* (37.5%) was, for the most part, due to the detection of the non-pathogenic *Y. enterocolitica* 1A strain by the comparator and reference methods, as opposed to the LiquidArray Gastrointestinal assay, which specifically detects the *ail* gene of the five pathogenic *Y. enterocolitica* strains (1B and 2–5). This was confirmed for four of five discrepant *Y. enterocolitica* results. Considering only pathogenic *Y. enterocolitica* detection, the sensitivity of LiquidArray Gastrointestinal in our dataset would be 75.0% (3/4 instead of 3/8; Table 5). This result emphasizes the superiority of the LiquidArray Gastrointestinal assay to detect clinically relevant *Y. enterocolitica* pathogens.

Other advantages of the LiquidArray Gastrointestinal assay compared to competitor assays are the broad spectrum of detected targets (25 targets covering up to 26 clinically relevant enteropathogens), its high-throughput capability (48 parallel samples processed per run within 5 h and with limited hands-on time) and the automated analysis allowing unambiguous result interpretation. The benefit of the extended panel was further evidenced by the need of seven comparator assays to cover the LiquidArray Gastrointestinal panel for this performance evaluation study. Finally, it should be noted that a low-throughput version of the LiquidArray Gastrointestinal assay has been recently validated, offering additional testing flexibility to the user. Despite these advantages and its good performance, the LiquidArray Gastrointestinal assay presents, like any other multiplex PCR assays, the disadvantages of being limited to the targets included on the panel and of detecting nucleic acids from living as well as from dead organisms. This emphasises the importance of implementing comprehensive syndromic panels in combination with conventional stool culture for an accurate diagnosis of active infections. When further combined with an efficient antimicrobial stewardship program, multiplex syndromic panels are associated with better patient outcomes and reduced healthcare costs [43,44].

The strengths of this study include the high number of enrolled samples, the combined use of comparator and reference methods (in case of discordant result) for the performance evaluation and the consideration of initial (and not after-repeat) indeterminate results for the analysis to adhere with the manufacturer’s instructions and thus prevent a bias in the performance analysis.

This study presents a few limitations, such as its single-centre design and the lack of sensitivity calculation based on real-life samples for very rare pathogens (*E. histolytica*, *P. shigelloides*, *V. cholerae*, *Vibrio* spp. and *C. cayetanensis*). Future studies should consider evaluating the performance of the LiquidArray Gastrointestinal assay in a multicentre setting, including sites with higher prevalence of these rare pathogens.

## 5. Conclusions

The LiquidArray Gastrointestinal assay showed an excellent diagnostic accuracy and a very low rate of invalid results, demonstrating its suitability for implementation in clinical routine. By allowing the simultaneous detection of a broad panel of clinically relevant enteropathogens, and owing to its high-throughput capability, the LiquidArray Gastrointestinal assay may lead to a rapid diagnosis of gastroenteritis, potentially improving patient management and disease control.

## Figures and Tables

**Table 1:** Comparator methods used to evaluate the clinical performance of LiquidArray^®^ Gastrointestinal VER 1.0.

LiquidArray^®^ Gastrointestinal	Comparator Methods	Reference Method in Case of Discordance
*Campylobacter* spp., EIEC/*Shigella* spp., *Salmonella* spp., *Vibrio* spp., *Yersinia enterocolitica*	Allplex GI-Bacteria(I) ^1^	BioFire^®^ FilmArray^®^ GI Panel ^2^
EAEC, *E. coli eae* gene, *E. coli* O157 gene, ETEC, Shiga toxin genes *stx1*/*stx2*	Allplex GI-Bacteria(II) ^1^	BioFire^®^ FilmArray^®^ GI Panel ^2^
Adenovirus 40/41, Astrovirus, Norovirus GI, Norovirus GII, Rotavirus, Sapovirus	Allplex GI-Viral ^1^	BioFire^®^ FilmArray^®^ GI Panel ^2^
*Cryptosporidium* spp., *Cyclospora cayetanensis*, *Entamoeba histolytica*, *Giardia lamblia* ^4^	Allplex GI-Parasite ^1^	BioFire^®^ FilmArray^®^ GI Panel ^2^
*Clostridioides difficile* toxins A and B	RealStar^®^ Clostridium difficile PCR Kit 2.0 ^3^	BioFire^®^ FilmArray^®^ GI Panel ^2^
*Plesiomonas shigelloides*, *Vibrio cholerae*	BioFire^®^ FilmArray^®^ GI Panel ^2^	None
*Ascaris* spp.	Allplex GI-Helminth(I) ^1^	None

^1^ Seegene, Seoul, South Korea. ^2^ BioFire Diagnostics, Salt Lake City, UT, USA. ^3^ Altona Diagnostics, Hamburg, Germany. ^4^ Also known as *Giardia duodenalis*.

**Table 2:** Patients’ and samples’ characteristics.

Study population, N (%)	1512 (100%)
Age in years, median (IQR)	51 (25–71)
Age class, N (%)	
<18 years old	302 (20.0%)
>18 years old	1182 (78.2%)
Unknown	28 (1.9%)
Sex, N (%)	
Male	714 (47.2%)
Female	771 (51.0%)
Unknown	27 (1.8%)
Stool sample type, N (%)	
Fresh, prospective	1179 (78.0%)
Frozen, archived	333 (22.0%)
Sample Bristol Stool Chart classification, N (%)	
Type 1	2 (0.1%)
Type 2	2 (0.1%)
Type 3	3 (0.2%)
Type 4	24 (1.6%)
Type 5	491 (32.5%)
Type 6	608 (40.2%)
Type 7	282 (18.7%)
Unknown	100 (6.6%)

Abbreviations: IQR, interquartile range; N, number of patients or samples.

**Table 3:** Negative and positive rates of LiquidArray Gastrointestinal test results.

All samples, N (%)	1512 (100%)
Negative samples, N (%) ^1^	983 (65.0%)
Positive samples, N (%) ^2^	529 (35.0%)
Detection type among positive samples, N (%)	
Mono-detections	386 (73.0%)
Co-detections ^3^	143 (27.0%)

^1^ Samples negative for all 25 pathogens; this includes 11 samples negative for 24 pathogens and indeterminate for one pathogen detection. ^2^ Samples with at least one positive detection. ^3^ Two-to-four pathogens detected per sample.

**Table 4:** Invalid rates of the LiquidArray Gastrointestinal vs. comparator results.

	Invalid Test Results	
Assay	Initial *n*/*N* (%)	After Repeat *n*/*N* (%)
**LiquidArray Gastrointestinal**		
PCR1	2/1512 (0.1%)	0/1512 (0.0%)
PCR2	5/1512 (0.3%)	0/1512 (0.0%)
**Comparators**		
Allplex GI-Bacteria(I) ^1^	36/1512 (2.4%)	10/1512 (0.7%)
Allplex GI-Bacteria(II) ^1^	25/1512 (1.7%)	5/1512 (0.3%)
Allplex GI-Viral ^1^	68/1512 (4.5%)	12/1512 (0.8%)
Allplex GI-Parasite ^1^	29/1512 (1.9%)	5/1512 (0.3%)
Allplex GI-Helminth(I) ^1^	0/110 (0.0%)	0/110 (0.0%)
BioFire^®^ FilmArray^®^ GI Panel ^2^	0/283 (0.0%)	0/283 (0.0%)
RealStar^®^ Clostridium difficile PCR Kit 2.0 ^3^	14/1510 (0.9%)	13/1510 (0.9%)

^1^ Seegene, Seoul, South Korea. For the very rare pathogen *Ascaris* spp. tested with Allplex GI-Helminth(I), only subsets of samples were tested (*n* = 110 tests performed), as described in the Materials and Methods section. ^2^ BioFire Diagnostics, Salt Lake City, UT, USA. For the very rare pathogens *P. shigelloides* and *V. cholerae* tested with BioFire FilmArray GI, only subsets of samples were tested (*n* = 283 tests performed), as described in the Materials and Methods section. It should be mentioned that although no invalid results were produced by BioFire FilmArray GI, five of the cartridges had to be discarded due to loss of vacuum prior to use. ^3^ Altona Diagnostics, Hamburg, Germany; 2 out of 1512 comparator data were missing (*n* = 1510 tests performed).

**Table 5:** Clinical performance of the LiquidArray Gastrointestinal assay relative to comparator and reference methods.

Pathogen	Sensitivity ^1^ *n*/*N* (%) [95% CI]	Specificity ^2^ *n*/*N* (%) [95% CI]	PPV ^3^ *n*/*N* (%) [95% CI]	NPV ^4^ *n*/*N* (%) [95% CI]	Likelihood Ratio (LR) ^5^
**PCR 1**					
*Campylobacter* spp.	144/148 (97.3%) [93.3–98.9]	1344/1352 (99.4%) [98.8–99.7]	144/152 (94.7%) [90.0–97.3]	1344/1348 (99.7%) [99.2–99.9]	164.4
*Clostridioides difficile* toxin A ^6^	60/66 (90.9%) [81.6–95.8]	1430/1430 (100%) [99.7–100]	60/60 (100%) [94.0–100]	1430/1436 (99.6%) [99.1–99.8]	- ^8^
*Clostridioides difficile* toxin B ^6^	59/65 (90.8%) [81.3–95.7]	1428/1432 (99.7%) [99.3–99.9]	59/63 (93.7%) [84.8–97.5]	1428/1434 (99.6%) [99.1–99.8]	325.0
*E. coli eae* gene	70/78 (89.7%) [81.0–94.7]	1409/1413 (99.7%) [99.3–99.9]	70/74 (94.6%) [86.9–97.9]	1409/1417 (99.4%) [98.9–99.7]	317.0
*E. coli* O157 gene	8/8 (100%) [67.6–100]	1498/1499 (99.9%) [99.6–100]	8/9 (88.9%) [56.5–98.0]	1498/1498 (100%) [99.7–100]	1499.0
EIEC/*Shigella* spp.	13/13 (100%) [77.2–100]	1489/1489 (100%) [99.7–100]	13/13 (100%) [77.2–100]	1489/1489 (100%) [99.7–100]	- ^8^
*Salmonella* spp.	30/30 (100%) [88.6–100]	1471/1472 (99.9%) [99.6–100]	30/31 (96.8%) [83.8–99.4]	1471/1471 (100%) [99.7–100]	1472.1
Shiga toxin genes *stx1*/*stx2*	22/24 (91.7%) [74.2–97.7]	1482/1483 (99.9%) [99.6–100]	22/23 (95.7%) [79.0–99.2]	1482/1484 (99.9%) [99.5–100]	1359.4
*Yersinia enterocolitica*	3/8 (37.5%) [13.5–69.4]	1494/1494 (100%) [99.7–100]	3/3 (100%) [43.9–100]	1494/1499 (99.7%) [99.2–99.9]	- ^8^
Norovirus GI	9/9 (100%) [70.1–100]	1491/1491 (100%) [99.7–100]	9/9 (100%) [70.1–100]	1491/1491 (100%) [99.7–100]	- ^8^
Norovirus GII	83/86 (96.5%) [90.2–98.8]	1414/1414 (100%) [99.7–100]	83/83 (100%) [95.6–100]	1414/1417 (99.8%) [99.4–99.9]	- ^8^
Rotavirus	25/33 (75.8%) [59.0–87.2]	1457/1463 (99.6%) [99.1–99.8]	25/31 (80.6%) [63.7–90.8]	1457/1465 (99.5%) [98.9–99.7]	184.7
*Cryptosporidium* spp.	14/14 (100%) [78.5–100]	1493/1493 (100%) [99.7–100]	14/14 (100%) [78.5–100]	1493/1493 (100%) [99.7–100]	- ^8^
*Entamoeba histolytica*	0/0 (-) ^8^ [-] ^8^	1507/1507 (100%) [99.7–100]	0/0 (-) ^8^ [-] ^8^	1507/1507 (100%) [99.7–100]	- ^8^
*Giardia lamblia* ^9^	25/28 (89.3%) [72.8–96.3]	1477/1479 (99.9%) [99.5–100]	25/27 (92.6%) [76.6–97.9]	1477/1480 (99.8%) [99.4–99.9]	660.3
**PCR 2**					
EAEC	14/25 (56.0%) [37.1–73.3]	1482/1482 (100%) [99.7–100]	14/14 (100%) [78.5–100]	1482/1493 (99.3%) [98.7–99.6]	- ^8^
ETEC	6/6 (100%) [61.0–100]	1498/1499 (99.9%) [99.6–100]	6/7 (85.7%) [48.7–97.4]	1498/1498 (100%) [99.7–100]	1497.0
*Plesiomonas shigelloides* ^7^	0/0 (-) ^8^ [-] ^8^	281/283 (99.3%) [97.5–99.8]	0/2 (0.0%) [0.0–65.8]	281/281 (100%) [98.7–100]	- ^8^
*Vibrio cholerae* ^7^	0/0 (-) ^8^ [-] ^8^	283/283 (100%) [98.7–100]	0/0 (-) ^8^ [-] ^8^	283/283 (100%) [98.7–100]	- ^8^
*Vibrio* spp.	1/1 (100%) [20.7–100]	1499/1499 (100%) [99.7–100]	1/1 (100%) [20.7–100]	1499/1499 (100%) [99.7–100]	- ^8^
Adenovirus 40/41	27/30 (90.0%) [74.4–96.5]	1465/1468 (99.8%) [99.4–99.9]	27/30 (90.0%) [74.4–96.5]	1465/1468 (99.8%) [99.4–99.9]	440.4
Astrovirus	12/13 (92.3%) [66.7–98.6]	1484/1487 (99.8%) [99.4–99.9]	12/15 (80.0%) [54.8–93.0]	1483/1485 (99.9%) [99.6–100]	457.5
Sapovirus	27/30 (90.0%) [74.4–96.5]	1464/1467 (99.8%) [99.4–99.9]	27/32 (84.4%) [68.2–93.1]	1464/1467 (99.8%) [99.4–99.9]	264.4
*Ascaris* spp. ^7^	4/5 (80.0%) [37.6–96.4]	105/105 (100%) [96.5–100]	4/4 (100%) [51.0–100]	105/106 (99.1%) [94.8–99.8]	- ^8^
*Cyclospora cayetanensis*	1/1 (100%) [20.7–100]	1506/1506 (100%) [99.7–100]	1/1 (100%) [20.7–100]	1506/1506 (100%) [99.7–100]	- ^8^

^1^ Sensitivity = TP/(TP + FN). ^2^ Specificity = TN/(TN + FP). ^3^ Positive Predictive Value = TP/(TP + FP). ^4^ Negative Predictive Value = TN/(TN + FN). ^5^ Likelihood Ratio = Sensitivity/(1-Specificity). ^6^ For *C. difficile* toxins A and B testing, 2 out of 1512 comparator data were missing (*n* = 1510 analysed paired samples; Figure 1). ^7^ For the very rare pathogens *P. shigelloides*, *V. cholerae* and *Ascaris* spp., only subsets of samples were tested with comparator methods, as described in the Materials and Methods section (*n* = 283 for *P. shigelloides* and *V. cholerae*, *n* = 110 for *Ascaris* spp.; Figure 1). ^8^ Not calculable. ^9^ Also known as *Giardia duodenalis*.

## Data Availability

The data presented in this study are available within the article and supplementary material.

## Supplementary Materials

The following supporting information can be downloaded at: https://www.mdpi.com/article/10.3390/diagnostics14212377/s1, Figure S1: LiquidArray^®^ Gastrointestinal VER 1.0 workflow; Figure S2: LiquidArray^®^ Gastrointestinal VER 1.0 result reporting; Table S1: LiquidArray^®^ Gastrointestinal VER 1.0 panel composition; Table S2: Number of samples with valid results in both the LiquidArray Gastrointestinal and comparator methods included in the clinical performance evaluation; Table S3: Indeterminate rates of the LiquidArray Gastrointestinal results among valid paired test results; Table S4: Confirmation of the clinical performance of the LiquidArray Gastrointestinal assay for the detection of rare pathogens (see Table 4) using contrived samples; Table S5: Co-amplification testing of the LiquidArray Gastrointestinal assay; Table S6: Cross-reactivity testing of the LiquidArray Gastrointestinal assay in duplicate PCR1 and PCR2 reactions using 59 commensal and non-target pathogens; Table S7: Precision testing of the LiquidArray Gastrointestinal assay.
