# Supplementary material for: Clinical Performance of the LiquidArray® Gastrointestinal VER 1.0 Assay in Patients with Suspected Gastroenteritis
Source: Diagnostics (Basel). 2024 Oct 25;14(21):2377. doi: 10.3390/diagnostics14212377 (PMC11544872; doi:10.3390/diagnostics14212377)
Supplement: Supplementary file 1 [file diagnostics-14-02377-s001.zip › diagnostics-3128055-supplementary.pdf]

Supplementary Files

Table S1. LiquidArray® Gastrointestinal VER 1.0 panel composition.

**PCR1 Pathogens**	**PCR2 Pathogens**
Bacteria	Bacteria
Campylobacter spp.	EAEC
Clostridioides difficile toxins A	ETEC
Clostridioides difficile toxins B	*Plesiomonas shigelloides*
E. coli eae gene (found in EPEC and EHEC)	*Vibrio cholerae*
E. coli O157 gene	*Vibrio* spp.
EIEC/*Shigella* spp.	
Salmonella spp.	
Shiga toxin genes *stx1*/*stx2* (found in STEC and EHEC)	
Yersinia enterocolitica	
Viruses	Viruses
Norovirus genogroup I (GI)	Adenovirus 40/41
Norovirus genogroup II (GII)	Astrovirus
Rotavirus	Sapovirus
Parasites	Parasites
Cryptosporidium spp.	*Ascaris* spp.
Entamoeba histolytica	*Cyclospora cayetanensis*
Giardia lamblia	

Abbreviations: EAEC, enteroaggregative *E. coli*; EIEC, enteroinvasive *E. coli*; EHEC, enterohaemorrhagic *E. coli*; EPEC, enteropathogenic *E. coli*; ETEC, enterotoxigenic *E. coli*; STEC, Shiga toxin-producing *E. coli*.

Table S2. Number of samples with valid results in both the LiquidArray Gastrointestinal and comparator methods included in the clinical performance evaluation.

Pathogen	Number of tests performed	Number of invalid LiquidArray tests after repeat	Number of invalid comparator tests after repeat	Number of analysed results [1]
PCR 1				
Campylobacter spp.	1512	0	10	1502
Clostridioides difficile toxin A [2]	1510	0	13	1497
Clostridioides difficile toxin B [2]	1510	0	13	1497
E. coli eae gene	1512	0	5	1507
E. coli O157 gene	1512	0	5	1507
EIEC/*Shigella* spp.	1512	0	10	1502
Salmonella spp.	1512	0	10	1502
Shiga toxin genes *stx1*/*stx2*	1512	0	5	1507
Yersinia enterocolitica	1512	0	10	1502
Norovirus GI	1512	0	12	1500
Norovirus GII	1512	0	12	1500
Rotavirus	1512	0	12	1500
Cryptosporidium spp.	1512	0	5	1507
Entamoeba histolytica	1512	0	5	1507
Giardia lamblia	1512	0	5	1507
PCR 2		0		
EAEC	1512	0	5	1507
ETEC	1512	0	5	1507
Plesiomonas shigelloides [3]	283	0	0	283
Vibrio cholerae [3]	283	0	0	283
Vibrio spp.	1512	0	10	1502
Adenovirus 40/41	1512	0	12	1500
Astrovirus	1512	0	12	1500
Sapovirus	1512	0	12	1500
Ascaris spp. [3]	110	0	0	110
Cyclospora cayetanensis	1512	0	5	1507

[1] Number of results valid for both LiquidArray Gastrointestinal and comparator methods that were included in the performance analysis. After repeat, none of the LiquidArray Gastrointestinal results were invalid, as opposed to comparator results, resulting in the indicated number of analysed paired valid results. [2] For *C. difficile* toxins A and B testing, 2 out of 1512 comparator data were missing (*n* = 1510 analysed paired samples; Figure 1). [3] For the very rare pathogens *P. shigelloides*, *V. cholerae* and *Ascaris* spp., only subsets of samples were tested with comparator methods, as described in the Materials and Methods section (*n* = 283 for *P. shigelloides* and *V. cholerae*, *n* = 110 for *Ascaris* spp.; Figure 1).

Table S3. Indeterminate rates of the LiquidArray Gastrointestinal results among valid paired test results.

Pathogen	Number of analysed results [1]	Indeterminate LiquidArray Gastrointestinal	
		Initial n/N (%)	After repeat n/N (%)
PCR 1			
Campylobacter spp.	1502	2/1502 (0.1%)	0/1502 (0.0%)
Clostridioides difficile toxin A [2]	1497	1/1497 (0.1%)	0/1497 (0.0%)
Clostridioides difficile toxin B [2]	1497	0/1497 (0.0%)	0/1497 (0.0%)
E. coli eae gene	1507	16/1507 (1.1%)	1/1507 (0.1%)
E. coli O157 gene	1507	0/1507 (0.0%)	0/1507 (0.0%)
EIEC/*Shigella* spp.	1502	0/1502 (0.0%)	0/1502 (0.0%)
Salmonella spp.	1502	0/1502 (0.0%)	0/1502 (0.0%)
Shiga toxin genes *stx1*/*stx2*	1507	0/1507 (0.0%)	0/1507 (0.0%)
Yersinia enterocolitica	1502	0/1502 (0.0%)	0/1502 (0.0%)
Norovirus GI	1500	0/1500 (0.0%)	0/1500 (0.0%)
Norovirus GII	1500	0/1500 (0.0%)	0/1500 (0.0%)
Rotavirus	1500	4/1500 (0.3%)	1/1500 (0.1%)
Cryptosporidium spp.	1507	0/1507 (0.0%)	0/1507 (0.0%)
Entamoeba histolytica	1507	0/1507 (0.0%)	0/1507 (0.0%)
Giardia lamblia	1507	0/1507 (0.0%)	0/1507 (0.0%)
PCR 2			
EAEC	1507	0/1507 (0.0%)	0/1507 (0.0%)
ETEC	1507	2/1507 (0.1%)	0/1507 (0.0%)
Plesiomonas shigelloides [3]	283	0/283 (0.0%)	0/283 (0.0%)
Vibrio cholerae [3]	283	0/283 (0.0%)	0/283 (0.0%)
Vibrio spp.	1502	2/1502 (0.1%)	0/1502 (0.0%)
Adenovirus 40/41	1500	2/1500 (0.1%)	0/1500 (0.0%)
Astrovirus	1500	0/1500 (0.0%)	0/1500 (0.0%)
Sapovirus	1500	1/1500 (0.1%)	0/1500 (0.0%)
Ascaris spp. [3]	110	0/110 (0.0%)	0/110 (0.0%)
Cyclospora cayetanensis	1507	0/1507 (0.0%)	0/1507 (0.0%)

[1] Valid for both LiquidArray Gastrointestinal and comparator methods (Table 4 and Table S2). [2] For *C. difficile* toxins A and B testing, 2 out of 1512 comparator data were missing (*n* = 1510 analysed paired samples; Figure 1). [3] For the rare pathogens *P. shigelloides*, *V. cholerae* and *Ascaris* spp, only subsets of samples were tested with comparator methods, as described in the Materials and Methods section (*n* = 283 for *P. shigelloides* and *V. cholerae*, *n* = 110 for *Ascaris* spp.; Figure 1).

Table S4. Confirmation of the clinical performance of the LiquidArray Gastrointestinal assay for the detection of rare pathogens (see Table 4) using contrived samples.

Pathogen	Positive Percent Agreement (PPA) *n*/*N* [1] (%)	Negative Percent Agreement (NPA) *n*/*N* [2] (%)
PCR1		
Entamoeba histolytica	5/5 (100%)	25/25 (100%)
PCR 2		
Plesiomonas shigelloides	5/5 (100%)	25/25 (100%)
Vibrio cholerae	5/5 (100%)	25/25 (100%)
Vibrio spp.	5/5 (100%)	25/25 (100%)
Cyclospora cayetanensis	5/5 (100%)	25/25 (100%)

[1] *n*/*N* is the ratio of the number of samples positive for the respective pathogen in the LiquidArray Gastrointestinal assay to the number of tested contrived (positive) samples. [2] *n*/*N* is the ratio of the number of samples negative for the respective pathogen in the LiquidArray Gastrointestinal assay to the number of tested negative samples.

Table S5. Co-amplification testing of the LiquidArray Gastrointestinal assay.

**Co-detected pathogens**	**Co-amplification result** ***n/N*** [1] **(%)**
PCR1	
Campylobacter spp. + *Salmonella* spp.	3/3 (100%)
Campylobacter spp. + EPEC	3/3 (100%)
Clostridioides difficile (A+B) + EPEC	3/3 (100%)
Clostridioides difficile (A+B) + STEC	3/3 (100%)
Clostridioides difficile (A+B) + Norovirus (GI + GII)	3/3 (100%)
Clostridioides difficile (A+B) + Rotavirus	3/3 (100%)
Norovirus GI + Norovirus GII	3/3 (100%)
Norovirus (GI + GII) + Rotavirus	3/3 (100%)
Norovirus (GI + GII) + EPEC	3/3 (100%)
EIEC/*Shigella* spp. + EPEC	3/3 (100%)
Giardia lamblia + EPEC	3/3 (100%)
PCR 2	
Adenovirus + Sapovirus	3/3 (100%)
Astrovirus + Sapovirus	3/3 (100%)

[1] *n/N* is the ratio of the number of samples positive for the respective co-detected pathogens in the LiquidArray Gastrointestinal assay to the number of tested positive samples (either cultured or clinical material combined prior to nucleic acids extraction).

Table S6. Cross-reactivity testing of the LiquidArray Gastrointestinal assay in duplicate PCR1 and PCR2 reactions using 59 commensal and non-target pathogens.

Species	Role	Source / Number	Form tested	Concentration tested
**Bacteria**				
Abiotrophia defectiva	Normal gut flora	CCUG 27807	Genomic DNA	6.8E+04 ge/rxn
Acinetobacter baumannii	Normal gut flora	DSM 30007	Microorganism	6.5E+05 CFU/ mL
Aeromonas hydrophila	Travellers' diarrhoea	LMG 21108	Genomic DNA	1.0E+07 ge/rxn
Alcaligenes faecalis	Normal gut flora	DSM 6174	Genomic DNA	9.0E+06 ge/rxn
Alistipes finegoldii	Normal gut flora	DSM 17242	Genomic DNA	4.1E+07 ge/rxn
Akkermansia muciniphila	Normal gut flora	DSM 22959	Genomic DNA	1.1E+07 ge/rxn
Arcobacter butzleri	Can cause diarrhoea	DSM 7301	Genomic DNA	1.0E+07 ge/rxn
Bacillus cereus	Can cause diarrhoea	DSM 31	Genomic DNA	3.8E+05 ge/rxn
Bacteroides fragilis	Normal gut flora	DSM 2151	Genomic DNA	1.0E+07 ge/rxn
Barnesiella intestinihominis	Normal gut flora	DSM 21032	Genomic DNA	2.3E+06 ge/rxn
Bifidobacterium bifidum	Normal gut flora	DSM 20215	Genomic DNA	2.0E+06 ge/rxn
Campylobacter fetus	Cross-reactivity	DSM 5361	Genomic DNA	4.2E+05 ge/rxn
Campylobacter concisus	Cross-reactivity	ATCC-BAA-1457D-5	Genomic DNA	5.2E+06 ge/rxn
Citrobacter amalonaticus	Normal gut flora	ATCC25405-D	Genomic DNA	1.6E+06 ge/rxn
Citrobacter koseri	Normal gut flora	ATCC-BAA-895D-5	Genomic DNA	6.3E+04 ge/rxn
Clostridium perfringens	Normal gut flora	DSM 2943	Genomic DNA	2.0E+05 ge/rxn
Clostridium septicum	Normal gut flora	DSM 7534	Genomic DNA	1.2E+05 ge/rxn
Clostridium tetani	Normal gut flora	ATCC19406-D3	Genomic DNA	2.7E+05 ge/rxn
Collinsella aerofaciens	Normal gut flora	DSM 3979	Genomic DNA	2.4E+05 ge/rxn
Edwardsiella tarda	Fish pathogen	ATCC15947-D3	Genomic DNA	1.7E+06 ge/rxn
Eggerthella lenta	Normal gut flora	DSM 15644	Genomic DNA	1.1E+07 ge/rxn
Enterobacter cloacae	Normal gut flora	DSM 30054	Microorganism	1.0E+05 CFU/mL
Enterococcus faecalis	Normal gut flora	DSM 2570	Microorganism	5.0E+05 CFU/mL
Escherichia coli	Normal gut flora	NCTC13476	Microorganism	1.0E+05 CFU/mL
Escherichia coli	Atypical EAEC	DSM 10974	Microorganism	3.7E+06 CFU/ mL
Faecalibacterium prausnitzii	Normal gut flora	ATCC-27766DQ	Genomic DNA	2.0E+05 ge/rxn
Fusobacterium varium	Ulcerative colitis	CCUG 35114	Genomic DNA	2.9E+07 ge/rxn
Gemella morbillorum	Normal gut flora	DSM 20572	Genomic DNA	6.0E+06 ge/rxn
Hafnia alvei	Normal gut flora	DSM 30163	Genomic DNA	6.8E+06 ge/rxn
Helicobacter pylori	Normal gut flora	ATCC700392D-5	Genomic DNA	2.0E+06 ge/rxn
Klebsiella pneumoniae	Normal gut flora	NCTC13442	Microorganism	1.0E+05 CFU/mL
Lactobacillus reuteri	Normal gut flora	DSM 20016	Genomic DNA	5.0E+06 ge/rxn
Listeria monocytogenes	Normal gut flora / foodborne pathogen	DSM 19094	Genomic DNA	3.1E+07 ge/rxn
Morganella morganii	Normal gut flora	DSM 30164	Genomic DNA	4.0E+06 ge/rxn
Parabacteroides distasonis	Normal gut flora	DSM 20701	Genomic DNA	4.1E+06 ge/rxn
Peptoniphilus asaccharolyticus	Normal gut flora	DSM 3032	Genomic DNA	5.4E+05 ge/rxn
Photobacterium damselae	Cross-reactivity	DSM 7482	Genomic DNA	7.7E+05 ge/rxn
Prevotella melaninogenicus	Normal gut flora	DSM 7089	Genomic DNA	5.0E+05 ge/rxn
Proteus mirabilis	Normal gut flora	DSM 4479	Genomic DNA	2.0E+06 ge/rxn
Providencia alcalifaciens	Can cause diarrhoea	DSM 30120	Genomic DNA	8.4E+06 ge/rxn
Pseudomonas aeruginosa	Normal gut flora	NCTC13437	Microorganism	1.1E+06 CFU/ mL
Staphylococcus aureus	Normal gut flora	DSM 20231	Microorganism	5.0E+05 CFU/ mL
Streptococcus agalactiae	Normal gut flora	DSM 2134	Genomic DNA	1.2E+06 ge/rxn
Veillonella parvula	Normal gut flora	CCUG 59474	Genomic DNA	5.4E+06 ge/rxn
Yersinia intermedia	Normal gut flora	DSM 18517	Genomic DNA	9.0E+06 ge/rxn
Yersinia pseudotuberculosis	Cross-reactivity	ATCC6904D-5	Genomic DNA	3.2E+06 ge/rxn
**Viruses**				
Adenovirus E (4)	Respiratory	0810070CFHI	Microorganism	1.1E+05 $TCID_{50}$/ mL
Coxsackievirus B3	Gastroenteritis	0810074CFHI	Microorganism	2.0E+06 $TCID_{50}$/ mL

Enterovirus 68	Respiratory	0810237CFHI	Microorganism	2.0E+03 $TCID_{50}$/ mL
**Parasites**				
Blastocystis hominis	Normal gut flora	ATCC-50177D	Genomic DNA	9.0E+05 ge/rxn
Dientamoeba fragilis	Normal gut flora	ATCC-PRA-3007SD	Genomic DNA	3.7E+05 ge/rxn
**Fungi**				
Candida albicans	Normal gut flora	ATCC-MYA-685	Genomic DNA	1.2E+04 ge/rxn
Candida parapsilosis	Normal gut flora	ATCC-22019	Genomic DNA	5.9E+04 ge/rxn
Candida tropicalis	Normal gut flora	ATCC-750	Genomic DNA	2.5E+04 ge/rxn
Candida glabrata	Normal gut flora	ATCC 2001	Genomic DNA	3.4E+05 ge/rxn
Candida krusei	Normal gut flora	ATCC 14243	Genomic DNA	2.7E+04 ge/rxn
Candida lusitaniae	Normal gut flora	ATCC 34449	Genomic DNA	2.6E+05 ge/rxn
Aspergillus versicolor	Environmental origin	DSM 1943	Genomic DNA	3.4E+03 ge/rxn
Saccharomyces cerevisiae	Foodborne	ATCC-18824	Genomic DNA	2.0E+04 ge/rxn

Table S7. Precision testing of the LiquidArray Gastrointestinal assay.

Reproducibility		
	**Variance (%)**	***P*-value** [2]
PCR 1 (*n* = 4928) [1]		
Between-operator precision	0.01	0.390
Between-cycler precision	0.01	0.365
Between-run (plate) precision	0.36	0.314
PCR 2 (*n* = 3620) [1]		
Between-operator precision	0.00	0.612
Between-cycler precision	0.04	0.074
Between-run (plate) precision	0.66	0.007
**Repeatability**		
PCR 1 (*n* = 4928) [1]		
Probability to get the same result, % [95% CI]	96.9% [96.4–97.4]	
PCR 2 (*n* = 3626) [1]		
Probability to get the same result, % [95% CI]	96.2% [95.5–96.8]	

[1] Number of measurements included in precision calculation. [2] ANOVA analysis of variance in combination with a linear mixed effects model. Abbreviation: CI, confidence interval.

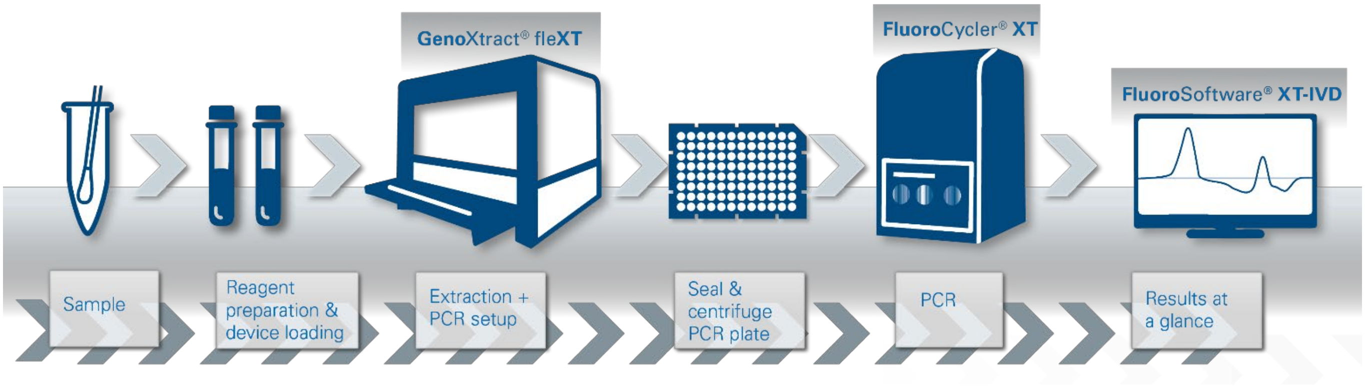

Figure S1. LiquidArray® Gastrointestinal VER 1.0 workflow. Stool samples are added to Stool Buffer and nucleic acids are extracted with the GXT96 X3 Extraction Kit on the GenoXtract® fleXT automated instrument. Real-time PCR and/or melting curves are generated on the FluoroCycler® XT thermocycler. Acquired fluorescence signatures are interpreted with the FluoroSoftware® XT-IVD software. Results are displayed automatically in the FluoroCycler® Report. LiquidArray® Gastrointestinal VER 1.0 allows the analysis and interpretation of 48 stool samples in about 5 hours.

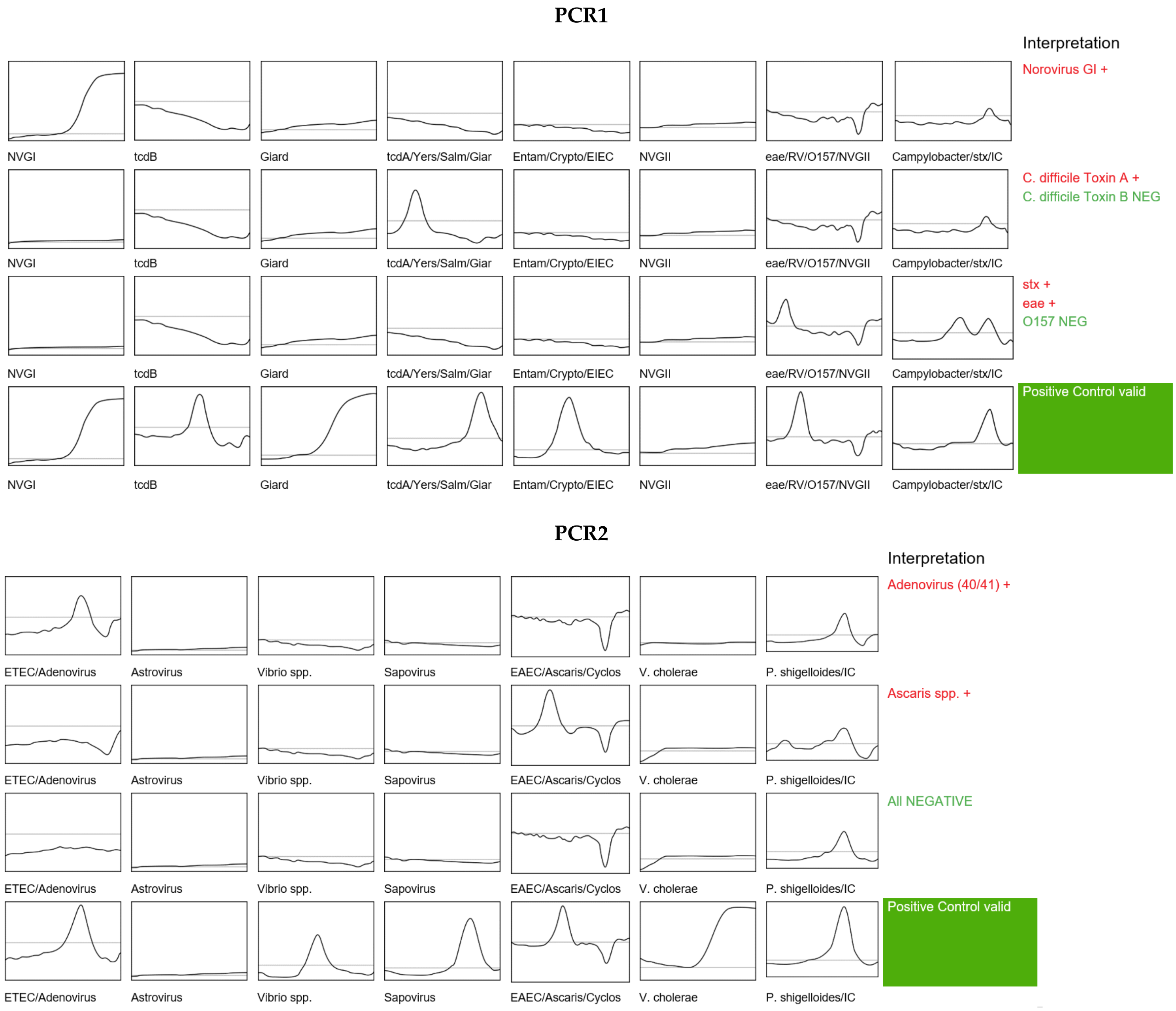

Figure S2. LiquidArray® Gastrointestinal VER 1.0 result reporting. Examples of fluorescence signatures for PCR1 and PCR2 testing, and their interpretation (positive/negative).
